# A Mathematical Investigation into the Design of Prefilters That Make Cameras More Colorimetric †

**DOI:** 10.3390/s20236882

**Published:** 2020-12-02

**Authors:** Yuteng Zhu, Graham D. Finlayson

**Affiliations:** School of Computing Sciences, University of East Anglia, Norwich NR4 7TJ, UK; g.finlayson@uea.ac.uk

**Keywords:** filter design, optimization method, camera sensors, colorimetry

## Abstract

By placing a color filter in front of a camera we make new spectral sensitivities. The Luther-condition optimization solves for a color filter so that the camera’s filtered sensitivities are as close to being linearly related to the XYZ color matching functions (CMFs) as possible, that is, a filter is found that makes the camera more colorimetric. Arguably, the more general Vora-Value approach solves for the filter that best matches all possible target spectral sensitivity sets (e.g., any linear combination of the XYZ CMFs). A concern that we investigate here is that the filters found by the Luther and Vora-Value optimizations are different from one another. In this paper, we unify the Luther and Vora-Value approaches to prefilter design. We prove that if the target of the Luther-condition optimization is an orthonormal basis—a special linear combination of the XYZ CMFs which are orthogonal and are in unit length—the discovered Luther-filter is also the filter that maximizes the Vora-Value. A key advantage of using the Luther-condition formulation to maximize the Vora-Value is that it is both simpler to implement and converges to its optimal answer more quickly. Experiments validate our method.

## 1. Introduction

When the spectral sensitivities of the RGB sensors found in a camera and the XYZ color matching functions (CMFs) [1] are exactly a linear transform apart, the camera is said to be colorimetric [2]. When this colorimetric condition is met, the RGBs measured by the camera—for all and any spectral stimuli—are the same linear transform from the corresponding XYZ coordinates. If a camera’s spectral sensitivities are a linear transform from the XYZ CMFs, it satisfies the so-called *Luther condition* [3,4].

Despite being suitable for color measurement, in general, cameras are rarely made to be colorimetric due to manufacturing and engineering considerations, including the need for low cost manufacturing and low-noise in the captured images [5]. Moreover, for cross-illuminant color measurement—where we wish to measure XYZs under different lights and then map the recorded tristimuli to a fixed reference illuminant, the best sensors are not linearly related to XYZ CMFs [6,7,8].

**Figure 1:**
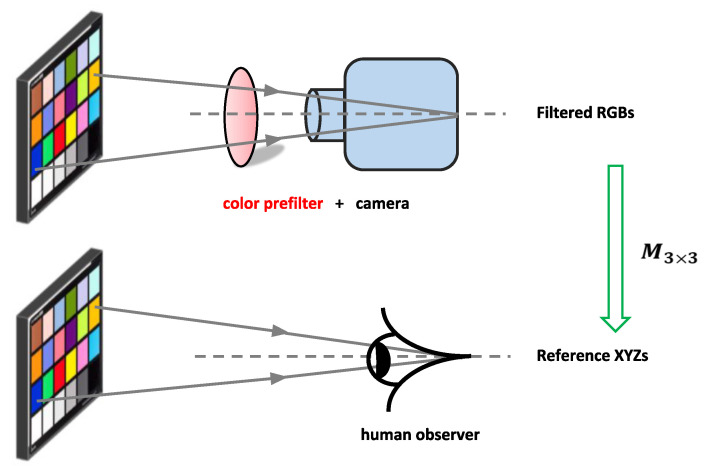
We propose to apply a prefilter for a digital camera such that its filtered RGBs can be best correct to the reference XYZ tristimuli.

It was recently proposed [9,10] that a color filter could be designed which, when placed in front of the camera, would make it more colorimetric, see Figure 1 (including for the cross-illuminant measurement case [10]). In Finlayson et al. [9], the filter is found that makes the camera best satisfy the Luther condition, that is, the filtered spectral sensitivities are as close as possible to being a linear 3×3 matrix correction from the XYZ CMFs. Curiously, the Luther-condition optimization is dependent on the target CMFs. If, for example, the best filter is found for the sRGB [11] sensor basis (a linear combination from the XYZ CMFs such as the cone mechanisms [12]), then a different optimal filter is found. That there are multiple optimal answers, perhaps, points to a weakness in the optimization statement?

In color science, the Vora-Value is a single number in the interval of [0, 1] which reports how close a camera’s spectral sensitivities are to the human visual subspace (HVS) [13]. When the Vora-Value is 1, this means the camera is colorimetric; when it is 0, it means the camera is completely unsuited for the color measurement task. The Vora-Value formula is, by construction, independent of the bases that describe the camera and the HVS [14]. In effect, the Vora-Value measures how close the vector spaces—respectively spanned by linear combinations of the camera spectral sensitivities and XYZ CMFs—are to one another. Finlayson and Zhu [15] employed the Vora-Value as an optimization criterion to solved for the optimal filter for a given camera.

From an algorithmic viewpoint, the Luther and Vora-Value optimizations are quite different from one another. The Luther condition optimization [9] is solved using a simple alternating least-squares procedure which converges quickly. The Vora-Value optimization [15], however, has a more complex derivation and is optimized by a gradient ascent approach. The Vora-Value optimization converges less quickly, though arguably to a better overall answer (because of the basis independence property of the Vora-Value).

The key result, which we present in this paper, is to prove that if we choose to optimize the Luther condition using not the XYZ CMFs but a linear combination which is orthonormal, then we are optimizing the Vora-Value. This means we can use the quicker-to-converge Alternating Least-Squares (ALS) optimization to find the best Vora-Value filter. Now, we recast the prior-art gradient-ascent Vora-Value approach so that we can use Newton’s method [16] (which includes 2nd derivatives) to speed up the optimization. Even compared to the faster Newton’s method, the simple ALS method has competitive performance, though it does converge a little more slowly.

However, an additional advantage of the ALS method is that it is easier to add constraints into the problem formulation than to do so for the Vora-Value approach. Indeed, prior work [17] has shown how we can bound both the smoothness of the derived filter and its transmission properties (e.g., that the filter has to transmit at least 40% of the light at every wavelength across the visible spectrum).

Experiments demonstrate that the Vora-Value optimized color filter can dramatically increase the colorimetric accuracy across a large set of commercial cameras.

The rest of the paper is structured as follows. In Section 2, we review the definition of the Luther condition and the Vora-Value. There, we introduce the concept of orthonormal basis which will underpin our optimization methods. In Section 3, we present the equivalence theorem relating the Luther- and Vora-Value optimizations. We also show how Newton’s method can be used to find the filter that maximizes the Vora-Value. Experimental results are reported in Section 4. The paper concludes in Section 5.

## 2. Background

### 2.1. Filter Design for Color Imaging

The problem of the design of spectral sensitivities for a color imaging system has been investigated in prior research. There, systems are assumed to have three RGB channels, such as color scanners and cameras, and the spectral sensitivities of the channels are designed to approximate the human visual system—subject to constraints such that the sensors can be manufactured—and to maximize the performance under well defined metrics such as Vora-Value [18], perceptual error metric [8], metamer mismatch body [19], and more general measures considering the imaging noise [13,20,21]. Vrhel and Trussell investigated the design of optimal filters under multi-illuminants and also in the presence of noise [6,22,23]. Interestingly, the best sensors that solve these problem are not linearly related to the XYZ CMFs [7].

A camera might capture multiple images each with a different filter placed in front of the camera [24,25] in order to obtain a greater than 3-dimensional signal (which is more easily mappable to colorimetric values). The filters can be selected from a group of commercially available filters [26,27,28,29,30,31]. Given known filters, an exhaustive search method can be used to select the one (or a set) that performs best [18,31]. Another possible way to increase the dimensionality of spectral capture is to use spectrally-tunable LED lights [32,33] to make multiple lights and to capture an image for each of these lights. Here, the lights perform an analogous role to the filters. However, whether we use commercially available filters or design lights with custom spectral power distributions, these filters and lights are not optimal. In other prior art, theoretically optimal filters are found through optimization [18,23,25,34] (though, these theoretical filters may not be manufacturable).

The method we propose here is much simpler. Given an off-the-shelf camera, we simply place a specially designed filter in front of the camera and then take a single-shot image. The design of such a prefilter is cast as an optimization problem. In this paper, we focus on finding the optimal color filter that makes a digital camera more colorimetric. Our approach is mathematical. We seek the best filter without worrying about whether we can fabricate the filter we design. However, in Section 3.5, we show how smoothness and transmittance constraints can be incorporated into the problem formulation. Not only does this allow us to control the optimization but these filters are plausibly easier to manufacture.

### 2.2. The Luther Condition

The Luther condition is as follows—if for all spectral stimuli, we would like a camera to be exactly colorimetric, that is, its RGB responses are a fixed linear transform from the corresponding XYZ tristimuli, then the camera’s spectral sensitivity functions must be a linear combination of the CIE XYZ CMFs [2].

Let Q=[r,g,b] and X=[x,y,z] denote respectively the spectral sensitivities of the camera and the CMFs of the human visual sensors. The columns of matrices Q and X represent the spectral sensitivity for each sensor channel and the rows represent the sensor responses at a sampled wavelength. Both matrices are in the size of n×3, where *n* is the number of sampling wavelengths across the visible spectrum (typically, n=31 when the visible spectrum running from 400 nm to 700 nm is sampled every 10 nm).

Mathematically, the Luther condition is written as

(1) X=QM,

where M is a 3×3 full rank matrix mapping the camera sensitivities to the human visual system. Equivalently we write:

(2) $${\mathrm{XT}}_{1}={\mathrm{QT}}_{2}M^{\prime },$$

where $T_{1}$, $T_{2}$ and $M^{\prime }$ are 3×3 full rank matrices. Clearly, given Equation (1), then $M^{\prime }=T_{2}^{-1}{\mathrm{MT}}_{1}$. That is, the Luther condition does not depend on the particular basis used; we can map camera sensitivities to XYZs, cone functions or any linear combination thereof. This is a key property that we will return to in Section 3.

If a camera satisfies the Luther condition, for any two color signals, f and g that integrate to the same RGB, the corresponding pair of XYZs must also be the same. That is,

(3) $$Q^{T}f=Q^{T}g\;\overset{M}{\Rightarrow }X^{T}f=X^{T}g$$

and thus are indistinguishable to the human observer. Readers are referred to  Reference [2] for more detail.

### 2.3. The Vora-Value

The Vora-Value is often used to measure how similarly a camera samples the spectral world compared to the human visual system. Mathematically, it measures the geometric closeness between the vector spaces spanned by the spectral sensitivity functions of a camera and a human observer. The Vora-Value is a number between 0 and 1, where 1 means the camera subspace shares the same vector space with the human visual subspace and therefore the camera RGBs can be corrected to the XYZ tristimulus values within a linear transform. Contrastingly, a Vora-Value of 0 means the two vector spaces are orthogonal to each other.

Given a camera sensor set Q and the trichromatic human visual sensors X, the Vora-Value is defined [18] as

(4) $$\begin{array}{c} \nu \left(Q,X\right)=\frac{1}{3}trace\left(Q{\left(Q^{T}Q\right)}^{-1}Q^{T}X{\left(X^{T}X\right)}^{-1}X^{T}\right), \end{array}$$

where the superscripts ${}^{T}$ and ${}^{-1}$ denote the matrix transpose and inverse, respectively, and trace() returns the sum of the elements along the diagonal of a matrix.

This representation can be written in a more compact way when we used the notation of matrix projector. The projector of a matrix, such as Q, is defined as

(5) $$P\left\{Q\right\}=Q{\left(Q^{T}Q\right)}^{-1}Q^{T},$$

thus we can rewrite the Vora-Value in a more compact way as

(6) $$\begin{array}{c} \nu \left(Q,X\right)=\frac{1}{3}trace\left(P\left\{Q\right\}P\left\{X\right\}\right), \end{array}$$

where P{Q} and P{X} denote the projection matrices respectively of the camera spectral responses and the human visual sensitivities, respectively.

Projector matrices often occur in least-squares regression. For example, finding x in the classic regression problem Ax≈b has a closed form solution. The linear combination of the columns of A that solves the least-squares regression is written as: $x={\left(A^{T}A\right)}^{-1}A^{T}b$, which makes the least-squares approximation $\mathrm{Ax}=A{\left(A^{T}A\right)}^{-1}A^{T}b$ (the projector of A multiplied by b).

### 2.4. Orthonormal Basis

**Figure 2:**
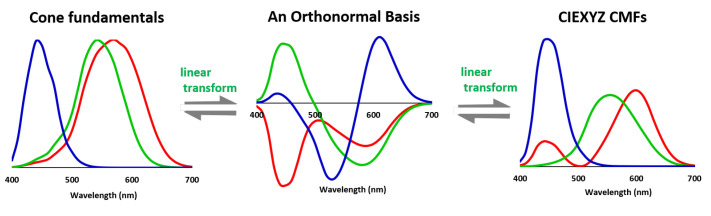
The cone fundamentals, a set of orthonormal basis and the CIEXYZ color matching functions (CMFs) are all linearly related, i.e., they can be converted to each other via a linear combination. All three sensitivity sets span the same vector space of the human visual system.

In an *n*-dimensional vector space, a basis is a set of *n* linearly independent vectors. Linear independence of the basis ensures that any vector in the vector space can be expressed as a linear combination of the basis vectors (see Figure 2). We say the basis spans the vector space. There are many bases for a vector space and, by definition, they are all linear transform apart.

Let $V=[v_{1},v_{2},v_{3}]$ denote a special linear combination of X that is orthonormal (see Figure 1), that is, V=XT where T is the linear mapping matrix. Orthonormality implies that its columns are unit vectors and also perpendicular to each other. Mathematically, we write $V^{T}V=I_{3}$ ($I_{3}$ is the 3×3 identity matrix). The orthonormal matrix V can be obtained by many methods, for example, the QR factorization [35].

By simple substitution into the matrix projector in Equation (5), we can express projector matrices (and the Vora-Value) in a simpler algebraic form:

(7) $$P\left\{X\right\}=P\left\{V\right\}={\mathrm{VV}}^{T}.$$

Denoting W as an orthonormal basis for the camera subspace spanned by Q, the simplified Vora-Value is written as:

(8) $$\begin{array}{c} \nu \left(W,V\right)=\frac{1}{3}trace\left({\mathrm{WW}}^{T}{\mathrm{VV}}^{T}\right). \end{array}$$

## 3. Methods and Algorithms

In previous work [9], it was shown how to find a filter that best satisfies the Luther condition under the assumption that we wish to find filtered camera sensors that best approximate XYZ CMFs. Further, the optimization was formulated using the Alternating Least-Squares paradigm. Below we reformulate the problem and make three important modifications. First we make an orthonormal basis set (linear combination) of the CMFs as the target. This step will help elucidate the similarity between optimizing a filter to best meet the Luther condition and to maximize the Vora-Value. Second, we recast the optimization as a gradient ascent optimization (and study the Newton formulations). Third, to ensure the well foundedness of the formulation—and to bound the shape of the filter—we add an additional regularization term that constrains the filter.

### 3.1. The Modified-Luther Condition Optimization

Physically, the effect of placing a transmissive filter, f (a 31×1 vector), in front of a camera can be modeled as diag(f)Q where diag() is a matrix operation that turns a vector into a diagonal matrix. To ease notation, we define F=diag(f) and write FQ. When we wish to extract f from F, we write f=ediag(F) (‘*e*’ signifies to ‘extract’ the diagonal).

In Equation (9) we mathematically formulate the filter-modified Luther-condition optimization:

(9) $$\underset{F,\;M}{arg min}\;\mu \left(F,\;M\right)={\;\|\mathrm{FQM}-V\|}_{F}^{2}+{\;\alpha ||\;F\;||}_{F}^{2},$$

where the subscript ${}_{F}$ denotes the Frobenius norm. Note that here we do not constrain the filter to be positive although a physical filter should have positive transmittance values. We remove this constraint because we found that, empirically, our method always returned an all-positive filter solution. Here we use the symbol μ to denote the objective function with the regularization term. Clearly, ${\left\|\mathrm{FQM}-V\right\|}_{F}^{2}$ is the core optimization: we wish to find F (the filter) such that the filtered sensitivities are approximately a 3×3 linear transform M from the orthonormalized CMFs, here V. The second term in the optimization is the squared norm of the filter where α≥0 is a penalty term defined by the user. As α becomes large, the optimization must effectively choose a filter whose norm is smaller. Small norm filters tend to be smoother and less jaggy. We say that the scalar α regularizes the optimization.

The regularization term in the optimization makes it tilt towards a filter with lower transmittance values over those with higher transmittances. Indeed, a filter that has a value of 1% at every wavelength has a lower norm than one which transmits 100% of the light at every wavelength. However, we argue that this is not a problem. Remember that in the core optimization, F and M are recovered up to a scaling factor, that is, αF and $\frac{1}{\alpha }M$ is an equally good solution. Viewed through the lens of this scaling indeterminacy, the filter that reflects 1% of light is equally efficacious as the one that transmits 100%. Pragmatically, because we know this scaling indeterminacy exists, we always scale the optimized filter so that its maximum transmittance value is 100%.

Importantly, the regularization term usefully pushes the optimization towards recovering smoother filters. A very jaggy filter with an average transmittance of (say) 50% has a much higher norm than one that has a uniform 50% transmittance across the spectrum. Jaggy filters are harder to manufacture so penalizing their discovery in the optimization makes sense.

In Section 3.5, we show how we can add more explicit constraints on the shape and transmittance properties of the filters that we design.

### 3.2. The Equivalence of the Modified-Luther and Vora-Value Optimizations

We begin by considering the special case of α=0 in Equation (9):

(10) $$\underset{F,\;M}{arg min}{\left\|\mathrm{FQM}-V\right\|}_{F}^{2}.$$

Suppose we are given the filter matrix F, then, employing the closed-form Moore-Penrose inverse solution to the least-squares [35], the best M is obtained

(11) $$M={\left({\left(\mathrm{FQ}\right)}^{T}\mathrm{FQ}\right)}^{-1}{\left(\mathrm{FQ}\right)}^{T}V.$$

Here, we see that the best linear mapping M in the Luther-condition optimization of Equation (9) is essentially a function of the unknown variable matrix F. By multiplying this newly solved M with FQ, we have

(12) $$\mathrm{FQM}=\mathrm{FQ}{\left({\left(\mathrm{FQ}\right)}^{T}\mathrm{FQ}\right)}^{-1}{\left(\mathrm{FQ}\right)}^{T}V=P\left\{\mathrm{FQ}\right\}V.$$

That is, we pre-multiply the orthonormal basis for the XYZs, V, by the projector for FQ (see Equation (5) for the definition of projector matrix).

Substituting into Equation (10), the optimization can be rewritten as

(13) $$\underset{F}{arg min}{\left\|(P\{\mathrm{FQ}\}-I)V\right\|}_{F}^{2},$$

where I is the identity matrix. Equation (13) has the advantage that the optimization depends only on the filter F.

**Theorem** **1.** *By minimizing $\underset{F}{arg min}{\left\|(P\{\mathrm{FQ}\}-I)V\right\|}_{F}^{2}$, we maximize v(FQ,X).*

**Proof** **of** **Theorem** **1.** We will use the following axioms:

1. $trace\left(A^{T}A\right)={\left||A|\right|}_{F}^{2}$
2. trace(AB)=trace(BA)
3. trace(A+B)=trace(A)+trace(B)
4. P{A}P{A}=P{A}, idempotency of projectors
5. $P\left\{A\right\}={\left(P\left\{A\right\}\right)}^{T}$, the projector is symmetric.
6. trace(P{A})=rank(A).

Using axiom 1, we rewrite the formula in Equation (13) as

(14) $$\underset{F}{arg min}{\left\|\;(P\{\mathrm{FQ}\}-I)V\;\right\|}_{F}^{2}\;=\underset{F}{arg min}trace\left(V^{T}{\left(P\left\{\mathrm{FQ}\right\}-I\right)}^{T}\left(P\left\{\mathrm{FQ}\right\}-I\right)V\right).$$

Now, we carry out some algebraic manipulation of the argument of Equation (14). Using axiom 2 for moving $V^{T}$ to the other side and known from Equation (7), $P\left\{X\right\}={\mathrm{VV}}^{T}$, we rewrite

(15) $${\left\|\;(P\{\mathrm{FQ}\}-I)V\;\right\|}_{F}^{2}\;=trace\left({\left(P\left\{\mathrm{FQ}\right\}-I\right)}^{T}\left(P\left\{\mathrm{FQ}\right\}-I\right)P\left\{X\right\}\right).$$

Manipulating the above expression using the properties of projector matrix (axioms 3 through 5), we obtain

(16) $$\begin{array}{cc} {\left\|\;(P\{\mathrm{FQ}\}-I)V\;\right\|}_{F}^{2} & =-trace(P\{\mathrm{FQ}\}P\{X\})+trace(P\{X\}). \end{array}$$

Using axiom 6, clearly in Equation (16), trace(P{X}) is a positive constant given the known matrix X. Therefore, we minimize the derived expression by maximizing trace(P{FQ}P{X}). Thus, we can write:

(17) $$\underset{F}{arg min}{\left\|\;(P\{\mathrm{FQ}\}-I)V\;\right\|}_{F}^{2}\;\equiv \;\underset{F}{arg max}trace\left(P\left\{\mathrm{FQ}\right\}P\left\{X\right\}\right).$$

From the definition of Vora-Value, we know $\nu \left(\mathrm{FQ},V\right)=\frac{1}{3}trace\left(P\left\{\mathrm{FQ}\right\}P\left\{X\right\}\right)$. We conclude:

(18) $$\underset{F}{arg min}{\left\|\;(P\{\mathrm{FQ}\}-I)V\;\right\|}_{F}^{2}\;\equiv \;\underset{F}{arg max}\nu \left(\mathrm{FQ},V\right).$$

☐

In summary, we have shown that a least-squares procedure that finds a filter that—in combination with a linear least-squares mapping (see Equation (10)–(13))—best fits an orthogonal basis of the color matching functions (i.e., minimizes the fitting error) must simultaneously maximize the Vora-Value.

### 3.3. Gradient Ascent for Solving the Optimization

So far we have concerned ourselves with formulating the filter design problem, that is, writing down what it is we wish to solve for. Now, we wish to present the mechanics of how we optimize for the filter. Given we have shown the equivalence for maximizing the Vora-Value and minimizing the modified Luther condition error, there is only one optimization at hand. The ALS method—for optimizing the Luther condition—is well described in Reference [10]. Henceforth we will concern ourselves with maximizing the Vora-Value (safe in the knowledge that the filter that is found also optimizes the modified Luther condition problem) in this section. Moreover, presenting the gradient method is a necessary step for us to present a much faster algorithm: the Newton’s method [16].

Since we are trying to maximize the Vora-Value, we will adopt a gradient ascent procedure. For a given filter, we can calculate its Vora-Value. The set of all Vora-Values can be thought of as a hilly landscape. By calculating the derivative (how the Vora-Value changes) when the filter is modified in certain directions, we can choose to take a small step in a direction that increases the Vora-Value.

By choosing to modify the filter step-wise—at each step seeking to go further up the hill—we modify the filter until we are on the hill top. Of course there is nothing in the gradient ascent method that guarantees that we will find an optimal solution to the filter design problem. At minimum, we will certainly, arrive at a filter that is better than the initial guess (e.g., the 100% do-nothing filter $f={\left[1,1,\cdots ,1\right]}^{T}$ when the filter is fully transmissive). And, as we present later, we find a very good filter to our problem.

Remember that the Vora-Value is defined to be the trace of the projector of the filtered sensitivities pre-multiplying the projector for the XYZ CMFs. So we wish to maximize:

(19) $$\begin{array}{c} \underset{F}{arg max}\;\nu \left(F\right)=\;\frac{1}{3}\;trace\left(P\left\{\mathrm{FQ}\right\}\;P\left\{V\right\}\right). \end{array}$$

We maximize ν in the usual calculus way by first finding the derivative of the Vora-Value function and then updating F by moving in the direction of the gradient (and in so doing we increase ν). It can be shown—details given in Reference [36]—that the derivative of ν calculated with respect to F is calculated as:

(20) $$\frac{\partial \nu }{\partial F_{jj}}=\;\frac{2}{3}\;{\left[\left(I-P\left\{\mathrm{FQ}\right\}\right)P\left\{V\right\}P\left\{\mathrm{FQ}\right\}F^{-1}\right]}_{jj}.$$

Equivalently, the gradient of the objective function in terms of the underlying filter vector—remember f (=ediag(F))—can be written as

(21) $$\nabla \nu \left(f\right)=\;\frac{2}{3}\;ediag\left(\left(I-P\left\{\mathrm{FQ}\right\}\right)P\left\{V\right\}P\left\{\mathrm{FQ}\right\}F^{-1}\right),$$

where the gradient with respect to the filter vector, ∇ν(f), is a 31×1 vector. It is evident that the gradient function has a very interesting structure: it is the diagonal of the product of three projection matrices multiplied by the inverse of the filter (at hand).

The simple Gradient Ascent algorithm for finding the filter solution of the optimization is shown below (see Algorithm 1). Note that we use the term ‘gradient ascent’ (but not the more familiar term ‘gradient descent’) because we are maximizing our objective function. The algorithm works by updating the current filter at step *k* to a new filter at step k+1. At step *k* we calculate the gradient and then update according to: $f^{k+1}=f^{k}+t^{k}\nabla \nu \left(f^{k}\right)$. The term $t^{k}$ is the step size (a small positive value) which controls how far the filter should move towards the direction of the gradient. The simplest approach would be to set $t^{k}$ to a small fixed number. But, in this case, the optimization will take a long time to converge. To make convergence quicker, we adaptively choose the stepsize. In our work we find the best step $t^{k}$ per iteration using the line search method. We refer the reader to Reference [37] for more details.

As we will see in the next section, even when we choose the step size judiciously, convergence is still relatively slow. A common technique to find the filter more quickly is the Newton’s method (which involves calculating 2nd derivatives). A naive implementation of the Newton’s method quickly runs into numerical stability problems and one way to side step these is to adopt the regularized formulation of the Vora-Value formulation. Remembering our theorem linking the Luther-condition method to the Vora-Value, we can rewrite the maximization where we re-include the penalty term on the norm of the filter as a minimization:

(22) $$\underset{F}{arg min}-3\nu \left(\mathrm{FQ},V\right)+{\;\alpha ||\;F\;||}_{F}^{2}.$$

It follows that the gradient function of the regularized optimization (denoted by μ in Equation (9)) as

(23) $$\nabla \mu \left(f\right)=-2\;ediag(\left(I-P\left\{\mathrm{FQ}\right\}\right)P\left\{V\right\}P\left\{\mathrm{FQ}\right\}F^{-1})+2\alpha f.$$

Clearly, ∇μ(f)=−3∇ν(f)+2αf. In the next section we present the Newton’s method for finding the regularized filter and use the corresponding gradient from Equation (23).
**Algorithm 1** Gradient Ascent for solving the optimization.1:$k=0,\;f^{0}=f^{initial}$2:**repeat**3:    Compute the gradient: $\nabla \nu (f^{k})$4:    Choose a step size: $t^{k}>0$5:    Update $f^{k+1}:=f^{k}+t^{k}\nabla \nu (f^{k}$)6:   k←k+17:**until**$max(|\nabla \nu \left(f^{k}\right)|)\leq \eta$8:**return**$f^{k}$.

### 3.4. Newton’s Method for Solving the Regularized Optimization

Often simple gradient ascent is slow to converge. A better way to tackle the optimization is to adopt the Newton’s method when the second derivative (i.e., the Hessian matrix) of the objective function can be computed. Newton’s method uses the curvature information (second derivative) of the objective function and generally is faster to converge, typically it has a quadratic convergence speed compared to linearly for gradient ascent/descent. When a neighborhood of the optimal solution is reached, often in just a few iterations a solution with adequately high accuracy can be achieved. Describing the details of how the Newton’s method works is beyond the scope of this paper—but see Reference [37] for a review—we will rather describe how it is used and report the Hessian we calculate for the filter design problem.

The Hessian for the filter design problem is a 31×31 matrix (assuming 31 sample wavelengths) and is denoted $\nabla^{2}\mu$. The term $\nabla_{ij}^{2}$ denotes the partial derivative $\frac{\delta^{2}\mu }{\delta f_{i}\delta f_{j}}$. Again with the derivation given in Reference [36], the Hessian for our problem is calculated as:

(24) $$\begin{array}{cc} \nabla^{2}\mu =\; & -2\left(P\left\{\mathrm{FQ}\right\}F^{-1}\right)\circ \left(\left(I-P\left\{\mathrm{FQ}\right\}\right)P\left\{V\right\}P\left\{\mathrm{FQ}\right\}F^{-1}\right)+\left(F^{-1}P\left\{\mathrm{FQ}\right\}F^{-1}\right)\circ \left((I-P\{\mathrm{FQ}\})P\{V\}\right)\\ \; & +\left(\left(I-2P\left\{\mathrm{FQ}\right\}\right)P\left\{V\right\}P\left\{\mathrm{FQ}\right\}F^{-1}\right)\circ \left(P\left\{\mathrm{FQ}\right\}F^{-1}\right)\;+\left(F^{-1}P\left\{\mathrm{FQ}\right\}P\left\{V\right\}P\left\{\mathrm{FQ}\right\}F^{-1}\right)\circ I+2\alpha I, \end{array}$$

where ∘ denotes the Hadamard product (or elementwise product) of two matrices. In the equation, the last term relates to the regularization term. Because we are adding a non-zero multiple, α, to the identity matrix, we prove in Reference [36] that this ensures the Hessian to be positive definite. Because the inverse of Hessian drives the Newton’s method, it is essential the Hessian has full rank.

Newton’s method for the problem of filter design is encapsulated in Algorithm 2. Here, given an initial guess $f^{initial}$, we choose a stepsize after calculating the gradient and Hessian. Again step-size is tackled using the backtracking line search procedure [16]. Later we update the filter solution by using its gradient and Hessian: $f^{k+1}:=f^{k}-t^{k}{\left[\nabla^{2}\mu \left(f^{k}\right)\right]}^{-1}\nabla \mu \left(f^{k}\right)$. The algorithm will keep refine the filter until a stopping criterion is met. In this paper, we simply use an empirically determined maximum iteration number (e.g., 1000) when the algorithm is well converged.
**Algorithm 2** Newton’s method for solving the optimization.1:$k=0,\;f^{0}=f^{initial}$2:**repeat**3:    Compute the gradient and Hessian: $\nabla \mu (f^{k})$ and $\nabla^{2}\mu \left(f^{k}\right)$4:    Choose a stepsize: $t^{k}>0$5:    Update $f^{k+1}:=f^{k}-t^{k}{\left[\nabla^{2}\mu \left(f^{k}\right)\right]}^{-1}\nabla \mu \left(f^{k}\right)$6:   k←k+17:**until** stopping criterion is satisfied8:**return**$f^{k}$

As we will see in the next section the Newton’s method converges much more quickly than gradient ascent. However, the cost of implementation is significant. Here the 31×1 gradient and 31×31 Hessian must be calculated at each step in the iteration. More onerously the inverse of the Hessian must also be calculated (a computationally non-trivial operation).

### 3.5. Adding Constraints to the Filter Optimisation

**Figure 3:**
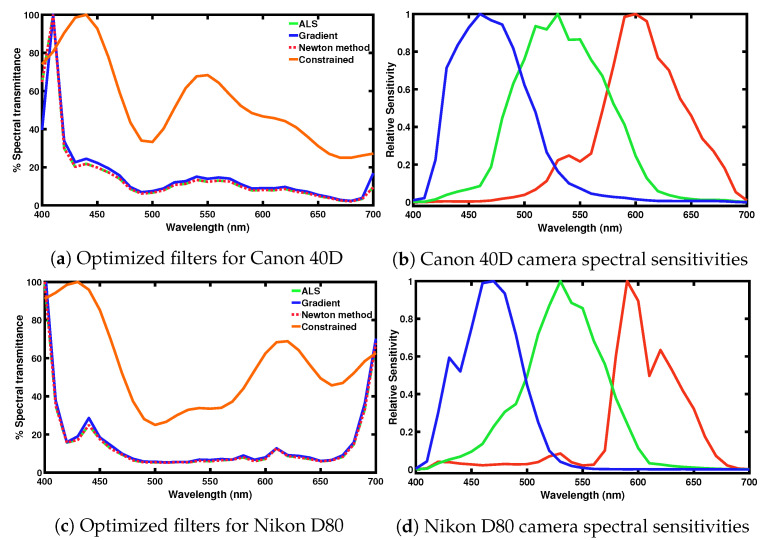
(**a**,**c**): Spectral transmittance of the derived optimized filters for a Canon 40D and a Nikon D80 DSLR cameras, respectively. The optimized transmissive filters are solved from the algorithms of the Alternating Least-Squares (solid green), gradient (solid blue) and Newton’s method (dotted red). The orange curves show the smoothness-constrained filters (that are described by the first 8 terms in a Cosine basis expansion) where they must transmit at least 25% of the light. (**b**,**d**): Camera spectral sensitivity curves of Canon 40D and Nikon D80 cameras.

By default the filter found by using the algorithms proposed in the previous subsections can be arbitrarily non-smooth (even with the regularization term) and it might also be very non-transmissive (see Figure 3). Non-smoothness can limit the feasibility of filter fabrication. Moreover, it is perhaps unsettling that we do not (and cannot) predict how filter smoothness relates to the regularization penalty term. As well as being smooth, we may also wish a color filter to transmit at least a lower bound percentage of the light that passes though. Indeed, a filter with too low transmittance values would perforce, have limited practical utility.

In this paper, we have shown how we can maximize the Vora-Value either by employing a gradient ascent algorithm or, equivalently, by adopting a simple Alternating Least-Squares (ALS) algorithm where we seek to find the filter such that the filtered sensitivities are close to being linearly transformable to an orthonormal basis of the human visual system sensitivity (e.g., a linear combination of the XYZ CMFs which are orthonormal). In previous work [17], we have shown how the ALS approach can be modified—using Quadratic Programming (QP) [38]—to incorporate smoothness and transmittance constraints.

While the QP solution is not the focus of this paper, we will include, for interest, an example of the constrained optimization in the next section. We refer the reader to Reference [17] where details of the constrained ALS formulation are presented.

## 4. Results

### 4.1. Optimal Filters

The optimal filters for a Canon 40D and a Nikon 80D DSLR cameras, derived from the modified Luther condition based optimization—where the target sensitivity set is the orthonormal vectors of the human visual space—are shown in Figure 3a,c respectively. In the figure, the maximum transmittance of the filters are normalized to 100%. The spectral sensitivity of the testing cameras are plotted in Figure 3a,d for reference. When solving for the filters, we use a regularization value of α=0.01 in the Newton’s method and α=0 in the ALS and gradient algorithms. On the left, we show the filter solutions derived from three solvers. Specifically, the optimal filters obtained using the ALS (in Reference [9]) in green, the gradient algorithm (Algorithm 1) in blue and the Newton’s method (Algorithm 2) in red. We can see that these three algorithms find very similar (almost the same) filter solutions for both cameras. So much so that you can only see the difference at the ends of the visible spectrum. Otherwise the graphs are on top of one another.

As readers may see in Figure 3a,c, the optimized filters have sharp ‘spiked’ peaks in the short wavelength range (and also the long wavelength range for Nikon D80 camera). This result at first glance may seem to be surprising but is actually not unexpected. Both cameras, Figure 3b,d, have little sensitivity at the ends of the visible spectrum and as consequence to locally change the shape of the effective sensitivities (filter multiplied by camera) necessitates spikes of the kind shown. Such spiky filters are surely difficult to manufacture (if they can be manufactured at all).

Moreover, these optimized filters may also be undesirable—even ignoring the spike— because they absorb most of the available light: they have low transmittance values across most of the visible spectrum. When a dark filter (it absorbs most of the light) is placed in front of a camera, we need to either increase the exposure time (or widen the aperture) or choose a higher ISO to obtain an image with the same light level as when no filter is applied.

For the ALS optimization—as discussed in Section 3.5—we can add constraints to the optimization and in so doing we can mitigate the spike and low transmittance problems. In orange, we show the derived optimal ALS filter transmittances when the filters are constrained both to transmit at least 25% of the light and to be a linear combination of the first 8 terms of a Cosine basis expansion.

### 4.2. Algorithm Performance

**Figure 4:**
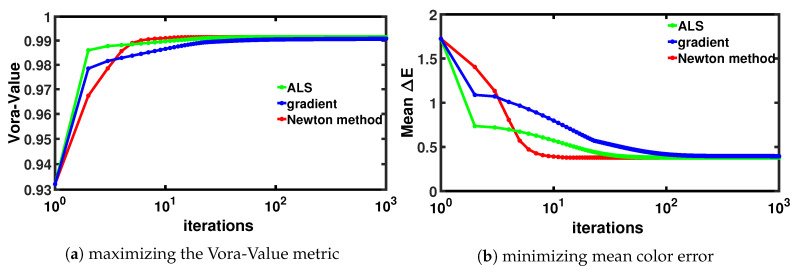
Algorithm convergence in terms of (**a**) Vora-Value and (**b**) mean $\Delta E_{ab}^{*}$.

Although these algorithms find highly similar filter solutions, there are significant differences in how they converge. In Figure 4, we show how many iterations it takes these three algorithms to converge (here we show the results of the Canon 40D camera). We evaluate their performance at each iteration in terms of two different error metrics—Vora-Value (in Figure 4a) and mean $\Delta E_{ab}^{*}$ (in Figure 4b). The color error metric $\Delta E_{ab}^{*}$ calculates the distance between a testing color and the reference color in a perceptually uniform CIELAB color space [39]. In our color measurement test, we use a collection of 102 illuminants and 1995 reflectance spectra [40] that represents the typical capturing scenarios. First, we calculate the RGBs (for the native camera without a filter and the camera sensitivities after filtering) and ground-truth XYZs of the reflectances under each illuminant using the physical modeling of color formation [10]. We linearly regress the RGBs to the XYZ counterparts before calculating the corresponding color difference $\Delta E_{ab}^{*}$ in CIELAB color space. The mean $\Delta E_{ab}^{*}$ used here is calculated by averaging the color error results over the reflectance set under all the illuminant conditions.

The performance of the ALS, gradient, Newton’s method algorithms are respectively shown in green, blue and red lines in Figure 4. We can see that the Newton’s method (red) converges slower in the first few iterations but quickly speeds up to arriving at the optimal solution. It takes only ∼10 iterations for a well-converged solution. Comparatively, for the same Vora-Value target, the numbers of iteration that ALS (green) and gradient (blue) algorithms need are, respectively, in the magnitude of $10^{2}$ and $10^{3}$ iterations. These two algorithms converge much slower, especially when approaching the optimal. We would like our readers recall that Newton’s method generally costs much more computationally, particularly for calculating the second derivative and its inverse. In contrast, the ALS method has a much simpler form which uses least-squares regression solved simply by the closed-form Moore-Penrose inverse [35].

### 4.3. Vora-Values and Color Measurement Experiment

Now we evaluate the performance of our optimized Vora-filters and compare to the prior art of Luther-filters [9]. The two filter design methods are noted as $f_{\mathrm{Vora}}$ and $f_{\mathrm{Luther}}$, respectively. Note that the result of $f_{\mathrm{Vora}}$ relates to the filter solved by using the ALS algorithm as it has simpler forms for implementation and obtains a filter solution with almost no difference to the other two algorithms. We also include the results of the native camera sensor, that is, without a filter (denoted **no-filter**) to establish the baseline results. The Vora-Value results are presented for both cameras Table 1. It is clear that with filters optimizing for the Luther condition, both deliver a higher Vora-Value (unsurprisingly, but a good test that our new Vora-Value method works).

From the table, we can see that the current filter $f_{\mathrm{Vora}}$—for Canon 40D—improves the Vora-Value to 0.991 from 0.932 for its unfiltered **no-filter** sensor sensitivities and higher than 0.986 by the prior art of the Luther-filter. Similar gain can also be seen for Nikon 80D camera on the right part of the table. Generally, a higher Vora-Value indicates greater similarity of the vector spaces spanned by the sensitivities of a camera and human visual system and therefore relates to substantially higher accuracy in color measurement [41].

Now we examine the performance of our derived filters in the color measurement experiment. We use the same data set and calculation process as described in Section 4.2.The color error statistics are shown in columns 3–7 for Canon and columns 9–13 for Nikon in Table 1. We can see that the filters from the current optimization performs the best (when no constraint is added). Compared to the baseline results, for both testing cameras, we can conclude that by using such optimized filters, we can effectively reduce the color errors by two thirds to three quarters. Compared to the $f_{\mathrm{Luther}}$, we see that a marginal improvement in Vora-Value score leads to a notable increase in color performance: a further 10% reduction.

Finally, for the much more transmissive and smooth filters (shown in Figure 3a,c)—see discussion in Section 3.5 for how these filters were derived—we include the calculated Vora-Value and colorimetric errors. While performance is not as good as for the unconstrained Vora-Value optimization, the results are still quite good and significantly better than using no filter.

**Figure 5:**
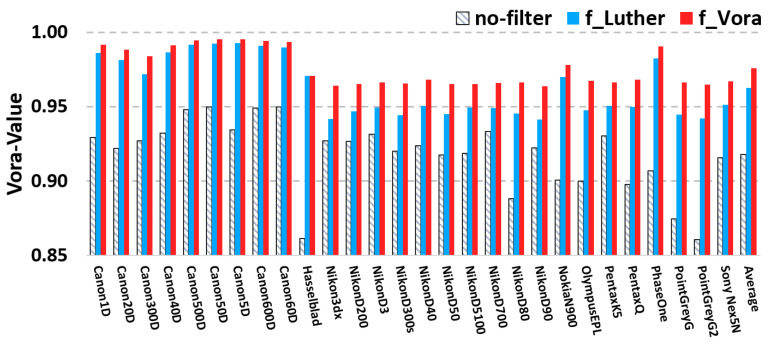
Results of Vora-Values for a group of 28 cameras: native sensitivities with no filter applied (neutral), the Luther filters optimized from the CMFs (blue) and the orthonormal-modified sensitivities (red). The overall averaged Vora-Values of the camera group are given in the last column.

Now, we extend the experiments to a much broader group of 28 digital cameras with known spectral sensitivity data set, including professional DSLRs, industrial and mobile cameras. These cameras have a clear deviation from the Luther condition, see Reference [42] for further detail. For each camera, we solve for the optimized filters given the corresponding spectral sensitivities. The Vora-Value performance for 28 cameras —with and without their optimized filters—are shown in Figure 5. The Vora-Value for the unfiltered native camera sensitivities are shown in chevroned grey bars and for the Luther-filters optimized sensitivities $f_{\mathrm{Luther}}$ in blue and the new $f_{\mathrm{Vora}}$ in red. It can be seen that the current optimized filters have the highest Vora-Value scores. The overall results, averaged across the whole camera group, are depicted in the rightmost bars. We see that, on average, the native Vora-Value is increased from 0.918 to 0.963 ($f_{\mathrm{Luther}}$) and 0.976 ($f_{\mathrm{Vora}}$) when the optimized filters are placed. Indeed, the current filter method $f_{\mathrm{Vora}}$ outperforms the prior art $f_{\mathrm{Luther}}$ for most cameras.

**Figure 6:**
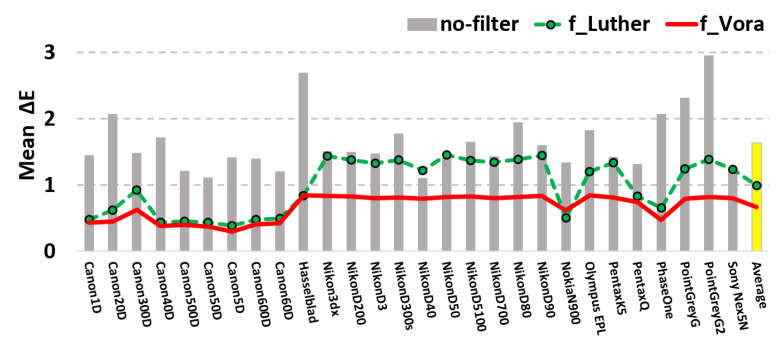
Mean $\Delta E_{ab}^{*}$ errors for 28 cameras. The grey-bars show the color errors for native cameras simply use color correction with no filter. The dashed green lines show the results by using the Luther-condition optimized filter. The results of the orthonormal-modified Luther optimized filters are plotted in solid red line.

Now we calculate the mean $\Delta E_{ab}^{*}$ error (using the same illuminant and reflectance data sets) for 28 cameras. For each camera we calculate the optimal $f_{\mathrm{Vora}}$ and $f_{\mathrm{Luther}}$ filters. Figure 6 summarizes the per camera mean $\Delta E_{ab}^{*}$ performance. Grey bars show the mean error performance of unfiltered color corrected RGBs for the 28 cameras. The dashed green and solid red lines record the performance of the best $f_{\mathrm{Luther}}$ and $f_{\mathrm{Vora}}$ filters, respectively.

Clearly by using optimized filters we have greatly improved color measurement for all 28 cameras and on average the performance increment is significant. Our current optimized filters, $f_{\mathrm{Vora}}$, also deliver significantly better color measurement performance compared with using the prior art of the Luther-condition optimized filters.

## 5. Conclusions

In this paper, we presented a unifying filter design method based on the underlying relation between the Luther condition and the Vora-Value. Mathematically, we prove that for the filter design problem, these two approaches can be well unified by using the orthonormal basis (a special basis set linearly transformed from the CMFs). That is, the optimal filter targeting at these two objectives can be simultaneously achieved. Hence, we can find an optimized Luther-filter that in the least-squares sense, also optimizes the Vora-Value. We have used three algorithms to solve for the best filter solution, that is, alternating least-squares (ALS), gradient ascent and Newton’s method; the latter of which has a faster convergence speed. We also find that the ALS approach has competitive performance compared to Newton’s method but has a much simpler formulation.

Experiments validate our method. For all cameras tested we find the Vora-Value is significantly increased compared to the cameras without filtering. In the color measurement test, we demonstrate that by using our optimized filters, we find the filtered sensitivities of digital cameras improve significantly in color accuracy compared to the prior arts.

## Figures and Tables

**Table 1:** Error statistics of the color corrected native camera with **no-filter**, the color corrected camera with the filters obtained from the sensitivity targets of the color matching functions $f_{\mathrm{Luther}}$ and of the orthonormal basis $f_{\mathrm{Vora}}$ for a Canon 40D DSLR and a Nikon D80 cameras.

	Canon 40D						Nikon 80D					
Method	Vora-	Color Errors $\Delta E_{\mathrm{ab}}^{*}$					Vora-	Color Errors $\Delta E_{\mathrm{ab}}^{*}$				
	Value	Mean	Median	95%	99%	Max	Value	Mean	Median	95%	99%	Max
**no-filter**	0.932	1.72	1.03	5.12	12.94	28.39	0.888	1.95	1.14	6.30	13.14	38.43
$f_{\mathrm{Luther}}$	0.986	0.44	0.22	1.48	3.19	8.77	0.945	1.39	0.87	4.49	7.35	11.43
$f_{\mathrm{Vora}}$	0.991	0.38	0.20	1.23	2.97	9.89	0.967	0.81	0.50	2.72	4.66	8.40
	with constraints						with constraints					
$f_{\mathrm{Vora}}$	0.985	0.56	0.43	1.27	3.66	12.86	0.947	0.90	0.59	2.76	5.00	8.96
